# Biventricular false tendons

**DOI:** 10.4322/acr.2023.432

**Published:** 2023-05-11

**Authors:** Stefano Tambuzzi, Guendalina Gentile, Federica Collini, Riccardo Zoja

**Affiliations:** 1 Università degli Studi di Milano, Laboratorio di Istopatologia Forense e Microbiologia Medico Legale, Sezione di Medicina Legale e delle Assicurazioni, Dipartimento di Scienze Biomediche per la Salute, Milano, Italy; 2 Università degli Studi del Piemonte Orientale Amedeo Avogadro, Dipartimento di Scienze della Salute, Novara, Italy

**Keywords:** Autopsy, Arrhythmias, Cardiac, Forensic Pathology, False tendon

False chordae tendineae or false tendons (FTs) are fibrous or fibro-muscular structures and sometimes also conduction tissue, blood vessels, and Purkinje cells that cross the ventricular cavities without connections to the valve cusps.^1^ Typically, they connect the free wall of the left ventricle or the papillary muscles and the ventricular septum, although they can also be found in the right ventricle.^2^ FTs must be distinguished from other entities, such as thickened ventricular trabeculae or ventricular masses. Most FTs are transverse and located at the apex; however, they may also be located in the apical, middle, or basal third in a diagonal or longitudinal orientation. They can be simple, with 1-2 insertion points, or branched with 3 or more insertion points.^3^ The exact prevalence of FTs in the general population varies widely, ranging from 0.4% to 83%.^3^ FTs appear to be higher in the pediatric age group.^4^ FTs are generally considered benign anatomic variants; however, they may be associated with varied entities, including precordial murmurs, repolarization abnormalities, preexcitation, mitral regurgitation, ventricular arrhythmias, and abnormal cardiac remodeling with systolic and diastolic dysfunction and dilatation of the left ventricle.^3-5^

FTs may be associated with electrocardiographic changes, especially in young sportspersons.^6^ Regarding ventricular arrhythmias, it has been suggested that they may be triggered by stretching the ventricular septum by FTs, resulting in increased automaticity. In this setting, it is suggested that FT may contain elements that are part of the cardiac excitation conduction system or cause excessive tension of Purkinje fibers in the cardiac septum. In addition, abnormal electrical impulses and re-entry circuits may be caused by fibrous tissue in FTs.^6^ However, the exact etiopathological mechanism of FT-induced cardiac changes remains unclear.^3^

**Figure 1:**
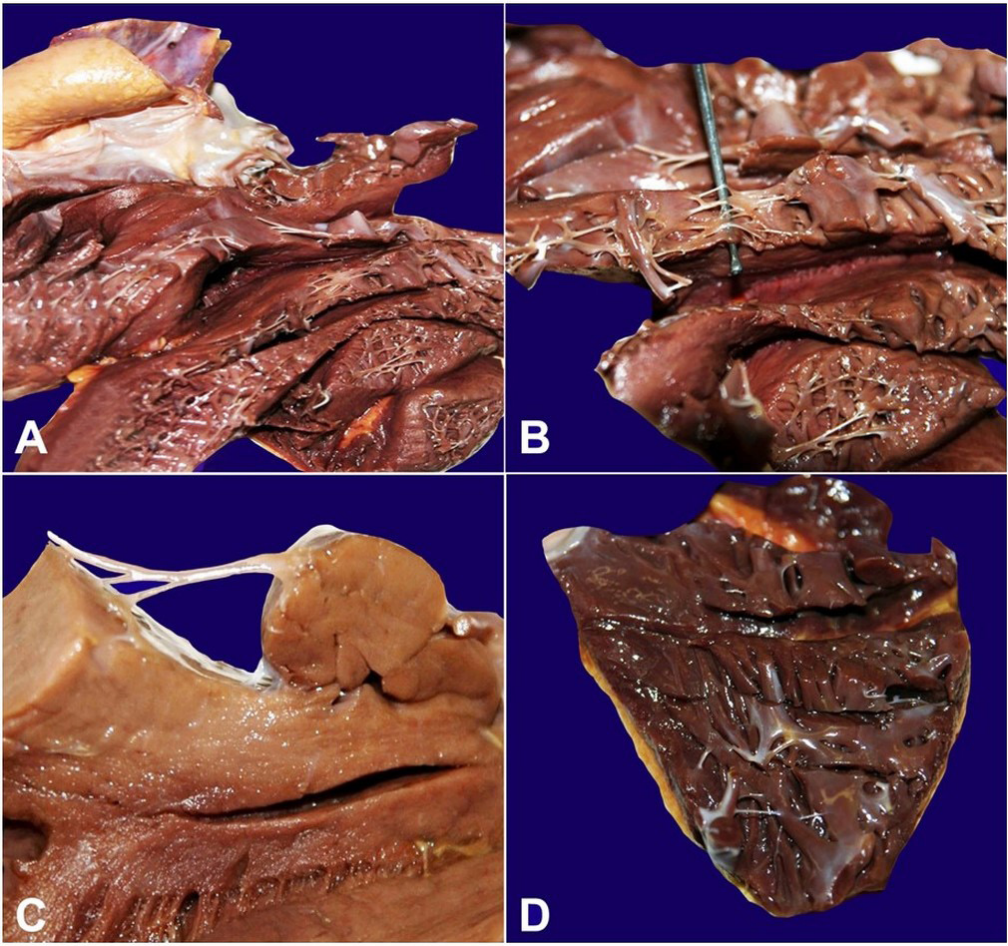
**A -** Macroscopic view of the heart, fixed in formalin and dissected. Anterior and posterior walls of the left ventricle are shown with evidence of numerous and diffuse foci of false tendons; **B -** Detail of the postero-lateral wall of the left ventricle with evidence of multiple false tendons that are predominantly transverse, have a branched morphology, and attach at multiple sites; **C -** Detail of a branched false tendon between the posterior papillary muscle and the endocardial surface of the left ventricle septal wall; **D -** Macroscopic view of the right ventricle, also characterized by the presence of multiple and diffuse foci of false tendons.

Figure 1 shows the heart of a 48-year-old Caucasian European man in good health, a bricklayer by trade, who died suddenly at home. At autopsy, the body measured 170 cm and weighed 65 kg (BMI 22.5 kg/m^2^). The heart was of normal shape, weighed 405 g, had a longitudinal diameter of 10.5 cm, a transverse diameter of 12 cm, and an anteroposterior diameter of 5 cm. No pathologic findings were noted in the epicardium or in the parietal and valvular endocardium. At the heart opening, the valves appeared normal, without vegetation or deformities. Specifically, the circumferences were as follows: at the mitral valve 3.2 cm, tricuspid valve 3.8 cm, aortic valve 2.2 cm, and pulmonary valve 2 cm. No signs of stenosis or valvular insufficiency were noted. Mild dilatation of the left ventricle with concomitant slight hypertrophy was observed. The thickness of the free wall of the left ventricle was: proximal third, 1.4 cm; middle third, 1.5 cm; distal third, 1.4 cm; and apex, 1.4 cm. The thickness of the septum was as follows: proximal third, 1.5 cm; middle third, 1.5 cm; and distal third, 1.4 cm. The maximum thickness of the right ventricle was 0.7 cm in the middle third. The myocardium was brownish, with discernible muscle fasciculations and normal consistency. In addition, numerous FTs were observed between the tip of the superior papillary muscle and the endocardial surface of the septal wall of the left ventricle, as well as additional irregularly arranged FTs foci in both ventricles. Finally, the coronary ostia and coronary arteries were free of obstruction and had intact walls.

Microscopically, the fibromuscular nature of the FTs was evident, with no other pathologic findings, neither in the heart nor the remaining viscera. At the end of the autopsy, death was attributed to an arrhythmic cardiac event.

This case was deemed of interest because the only finding was the presence of multiple biventricular FTs. These findings are rarely observed and reported in the literature and have been described almost exclusively by diagnostic imaging in the clinical setting. However, in this brief report, explanatory macroscopic images of FTs still in situ are reported, helping to fill a gap in the literature. Also, from another perspective, references in the literature to the significance of FTs sometimes need to be revised. Our case was a normal-weight white European male (BMI 22.5 kg/m^2^) with a heart of normal shape and weighing 405 g, characterized by multiple biventricular FTs foci, mild dilatation of the left ventricle, and slight hypertrophy. There were no other pathologic findings, either macroscopic or microscopic. The mild dilatation of the left ventricle was a finding consistent with the presence of multiple FTs, as reported in the literature.^3,5^ The left ventricular hypertrophy was of low intensity and was consistent with the man's physical activity as a bricklayer. The weight of the heart deserves special attention. Although it was slightly over 400 g, this weight is within the normal range for Caucasian men, given the height and BMI. These data were compared with the forensic study that examined the weight of organs in 684 autopsies of Caucasian adults, excluding all individuals who showed macroscopic signs of disease or histological abnormalities.^7^ It was found that the heart weight in the general population of healthy Caucasian men was 365 g +/- 71. With a height of 165-175 cm, the heart weight was 360 g +/- 75; with a BMI between 22 and 24, the heart weight was 370 g +/- 75. Thus, this case was not an enlarged heart. We faced a situation where no evidence could be interpreted as a definite cause of death. As already reported, the association of multiple FTs, left ventricular dilatation, and sudden arrhythmic events were acknowledged in the literature. It is noteworthy that in our case, there were no other pathological conditions that could trigger cardiac arrhythmias, such as valvular dysfunction, cardiomyopathies (including obesity-related diseases), cardiac thrombi, myocardial fibrosis, and heart failure, were ruled out. Notably, the extent of left ventricular dilatation observed was inconsistent with heart failure. Numerous reports suggest that FTs significantly impact cardiac electromechanical events.^2^ All of this has led us to believe that the FTs in the present case most likely played an active role in the death and thus were not mere bystanders. However, to date, no robust population-based evidence of an association between FTs and increased cardiac morbidity and mortality.^3^ This gives rise to an apparent paradox that poses considerable interpretive difficulties in situations like our case, where there were no other pathological findings and plausible causes to explain the death. This criticality can be overcome by considering that very few studies on this subject do not allow uncritical generalization. Therefore, precisely because of the possible clinical implications, each case in which FTs are documented should be analyzed individually while waiting for further research to help shed light on this exciting and still partially unexplored phenomenon.
